# Healthcare students’ attitudes towards patient centred care: a systematic review with meta-analysis

**DOI:** 10.1186/s12909-022-03371-1

**Published:** 2022-04-27

**Authors:** Geronimo Bejarano, Ben Csiernik, James J. Young, Kent Stuber, Joshua R. Zadro

**Affiliations:** 1 Michael & Susan Dell Center for Healthy Living, The University of Texas Health Science Center at Houston (UTHealth), 1616 Guadalupe Street, Austin, TX 78702 USA; 2 Canadian Memorial Chiropractic College, Toronto, Canada; 3 Center for Muscle and Joint Health, University of Southern Denmark, Odense, Denmark; 4 Sydney School of Public Health, Faculty of Medicine and Health, Institute for Musculoskeletal Health, The University of Sydney and Sydney Local Health District, New South Wales, Australia

**Keywords:** Patient centred, Students, Patient-practitioner orientation scale

## Abstract

**Background:**

Patient centred care is commonly recommended in clinical practice guidelines to improve patient outcomes and reduce healthcare costs. Identifying measurement tools used to assess healthcare students’ attitudes towards patient centered care and determining their attitudes is the first step to ensuring patient centred care is provided in the future. The primary aim of this review was to describe the measurement tools used to assess healthcare students’ attitudes towards patient centred care. The secondary aim was to quantify healthcare students’ attitudes towards patient centred care.

**Methods:**

An electronic database search was conducted in MEDLINE, EMBASE, CINAHL from inception until March 1, 2021, with combined terms relating to ‘patient centred care’, ‘attitudes’, and ‘healthcare students’. Studies that quantitatively assessed healthcare students’ attitudes towards patient centred care were included. Measurement tools used in the included studies were qualitatively described. Meta-analysis was conducted to quantify healthcare students’ attitudes towards patient centred care and assess the respective influence of gender, profession, and study geographical location on healthcare students’ attitudes towards patient centred care.

**Results:**

The electronic search identified 3948 total studies. One hundred twenty-nine full texts were screened, and 49 studies were included. There were 16 measurement tools used to assess healthcare students’ attitudes towards patient centered care. Most studies (53%, *n* = 26) used the Patient-Practitioner Orientation Scale (PPOS) to assess patient centered care. Meta-analyses of 20 studies with 26 total groups resulted in a pooled mean PPOS score of 4.16 on a 0–6 scale (95% Confidence Interval [CI]: 3.95, 4.37), indicating low attitudes towards patient centered care. Additional analyses found that women have significantly higher attitudes towards patients centred care than men (pooled effect 0.14 [95% CI: 0.05, 0.23], *n* = 8 studies) and mean PPOS scores appear similar among sub-groups of only medical students (pooled mean 4.13, 95% CI: 3.85, 4.42, *n* = 13 studies) and only American healthcare students (pooled mean 4.49, 95% CI: 4.35, 4.64, *n* = 5 studies).

**Conclusions:**

Several different measurement tools have been used to assess healthcare students’ attitudes towards patient centred care, but the most commonly used is the PPOS. Our results indicate that healthcare students have low attitudes towards patient centred care. Future studies should evaluate if attitudes towards patient centred care can be improved during healthcare education.

**Supplementary Information:**

The online version contains supplementary material available at 10.1186/s12909-022-03371-1.

## Background

Patient centred care occurs when healthcare providers are respectful of and responsive to patient preferences, needs and values, and ensures patient values guide all clinical decisions [1]. Patient centred care is multi-dimensional. For example, Mead an Bower [2] describe patient centred care as having five dimensions including a biopsychosocial perspective, the patient as a person, sharing power and responsibility, the therapeutic alliance, and the doctor as a person.

Using a patient centred care approach to deliver healthcare has been shown to reduce healthcare costs while improving patient outcomes [3, 4]. Unfortunately, not all healthcare professionals have positive attitudes towards patient centred care and attitudes vary between specialties [5]. Ensuring healthcare students have positive attitudes towards patient centred care is an important starting point to increase the number of healthcare professionals providing patient centred care. However, previous studies assessing healthcare students’ attitudes towards patient centred care have found mixed results. Some show that a large proportion of healthcare students have positive attitudes towards patient centred care, [6] while others show the opposite [7].

One possible explanation for these inconsistent findings is variation in the measurement tools used to assess attitudes towards patient centred care (e.g. Patient-Practitioner Orientation Scale [PPOS], Doctor-Patient Scale) [8, 9]. Understanding the different measurement tools used to assess healthcare students’ attitudes towards patient centred care is an important first step towards summarizing the available evidence on healthcare students’ attitudes towards patient centred care. Therefore, the primary aim of this study was to describe the measurement tools used to assess healthcare students’ attitudes towards patient centred care. Secondary aims were to quantify healthcare students’ attitudes towards patient centred care and assess the respective influence of gender, profession, and study geographical location on healthcare students’ attitudes towards patient centred care.

## Methods

This systematic review has been reported according to the Preferred Reporting Items for Systematic Reviews and Meta-Analysis Protocols (PRISMA) [10] and preregistered on Open Science Framework [11]. The PRISMA checklist is provided in Appendix 1.

### Search strategy

An electronic database search strategy was developed with a health sciences librarian and searches were conducted in MEDLINE, EMBASE, CINAHL from inception until March 1, 2021, with no language restriction. The search strategy and search terms were informed by previous reviews on patient centred care [12] and healthcare students [13]. Our search strategy combined terms relating to ‘patient centred care’, ‘attitudes’, and ‘healthcare students’ and was designed to capture studies investigating healthcare students attitudes towards patient centred care as per our preregistered protocol. The full MEDLINE search strategy is available in Appendix 2. Forward citation tracking was performed in Web of Science. All studies identified by our search strategy were retrieved and managed using Covidence systematic review software (Veritas Health Innovation, Melbourne, Australia).

### Study eligibility criteria

Studies that quantitatively assessed healthcare students’ (e.g. physical therapy, chiropractic, medicine, nursing, dentistry, etc.) attitudes towards patient centred care were included. Studies that measured mixed student and professional populations were included however, only if it was possible to extract data for students separately. Studies were not excluded based on language or type of measurement, provided it was quantitative. Qualitative studies and studies including only qualified health professionals were excluded.

### Study selection

Study selection was conducted in two phases: (I) the title and abstract review phase, and (II) the full text review phase. If a paper met inclusion criteria in phase (I), the full text was retrieved and reviewed for potential inclusion. Two reviewers (GB and BC) conducted title and abstract selection and full text review independently. Any disagreements were resolved by discussion or consultation with a third reviewer (JJY).

### Data extraction

Two reviewers (GB and BC) independently extracted individual study characteristics. Demographic data extracted included: author name, title, date of publication, journal, location of study, year of study completion, sample size and student characteristics (age, sex, profession). Data extraction items for study aims included: name of measurement tool and subscales, exact construct, number of items, and scoring for patient centred care measures (mean and standard deviation [SD] median interquartile range [IQR], author defined proportion of students who have positive attitudes towards patient centred care), and scoring across different sub-groups (e.g. based on age, sex, profession type). Any discrepancies were resolved by discussion between the two reviewers. Study authors were contacted when relevant data was not reported. In our protocol, we planned to extract effect measures (Odds Ratios, Risk Ratios or correlation coefficients) and measures of variability for associations between various predictor variables (e.g. age, sex, profession type) and attitudes towards patient centred care. However, no included studies reported this data.

### Risk of bias/study quality assessment

The methodological quality of included studies was assessed independently by two reviewers (GB and BC) using a modified version of the Downs and Black checklist (Appendix 3). We modified the original 27-item Downs and Black checklist [14] and selected 10 items that were relevant to studies assessing attitudes towards patient centred care. Selection of items to include in the modified Downs and Black checklist was decided by consensus between study authors prior to conducting the search. The individual studies were scored from 0 to 10 based on reporting clear objectives, outcomes, characteristics of included patients, findings, estimates of the random variability, actual probability values, recruitment and sample characteristics suggesting representativeness, appropriate statistical tests, and accurate outcome measures. A detailed description of the modified Downs and Black checklist is provided in Appendix 3. Any disagreements between the two reviewers were resolved through discussion.

### Data analysis

Characteristics of measurement tools used to assess attitudes towards patient centred care (e.g., name of tool, measurement construct, subscales, number of items) were qualitatively summarized. Quantitative data on attitudes towards patient centred care (mean (SD) or n (%)] was pooled when studies were considered sufficiently homogenous in terms of population and measure used to assess attitudes towards patient centred care. Meta-analysis was performed using the inverse-variance method with the Hartung-Knapp adjustment for random effects models [15]. Statistical heterogeneity was assessed using the I^2^ statistic [16]. The I^2^ statistic was interpreted as might not be important (0% to 40%), may represent moderate heterogeneity (30% to 60%), may represent substantial heterogeneity (50% to 90%), or considerable heterogeneity (75% to 100%) [17]. Analyses of factors that may influence healthcare students attitudes towards patient centred care were conducted on available variables (sex, medical students only, and United States [U.S.] medical students only) to explore whether any observed heterogeneity was due to differences in sex, profession, or geographical location across studies. Meta-analysis was conducted using R statistical software (https://www.r-project.org).

## Results

**Fig.1:**
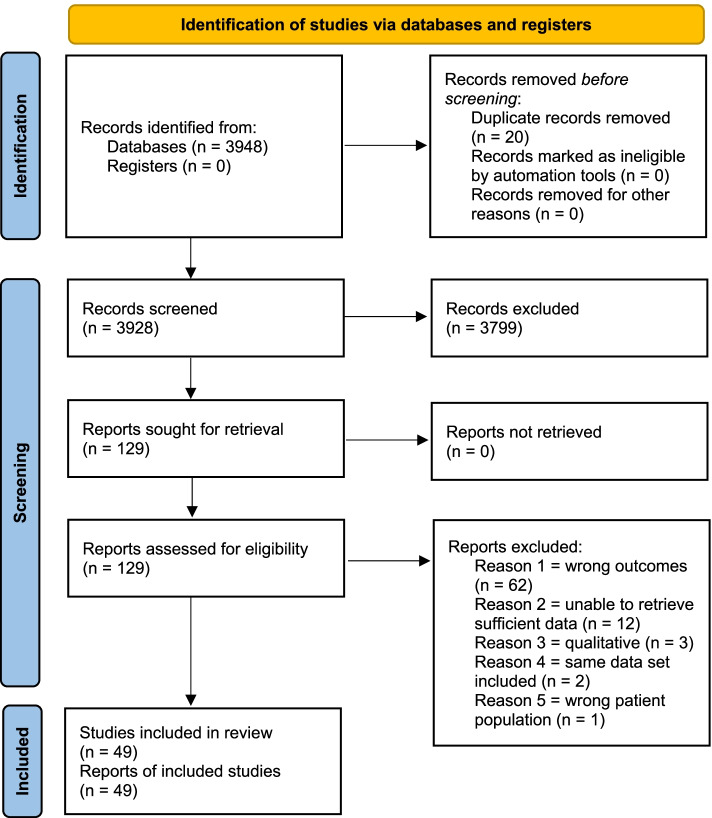
PRISMA flow chart of the records and study selection process

The initial electronic database search identified 3948 potentially eligible studies. After removing duplicates and screening studies by title and abstract, 129 potentially eligible studies for inclusion were considered and their full text was retrieved. After full text screening, 49 studies [6–9, 18–62] met the inclusion criteria and were included in the review with 20 studies (*n* = 8050) [6, 7, 9, 18, 25, 26, 28–32, 36, 39, 43, 45, 46, 49, 52, 54, 55] providing sufficient data for meta-analysis. Two studies were conducted using the same data set as other included studies therefore, we only used data from the original report in our meta-analysis [63, 64]. The PRISMA flowchart of studies through the review is provided in Fig. 1.

The authors of twelve studies were contacted for additional data and were ultimately excluded due to inability to retrieve data needed to determine whether they assessed healthcare students attitudes’ towards patient centred care [65–76].

### Characteristics of included trials

The 49 included studies used 16 different measurement tools to investigate healthcare students’ attitudes towards patient centred care, with sample sizes ranging from 32 to 3191 students. The majority of studies assessed U.S. healthcare students’ attitudes (40.8%) followed by United Kingdom healthcare students (8%). A comprehensive description of each study is provided in Table 1. Twenty-six studies (53%) used the PPOS measurement tool while three different modified versions of the PPOS were used in one study each. The Readiness for Interprofessional Learning Scale (RIPLS), Doctor-Patient Scale, and Interprofessional Attitudes Scale (IPAS) were each used in five, four, and two studies, respectively. The Health Beliefs Attitudes survey, Nelson-Jones and Patterson Counsellor Attitude scale, Patient-Centredness Multi-Choice Questionnaire, and Tucker-Culturally Sensitive Health Care Inventory Provider form were each used in one study. Five studies used measurement tools with no name reported. A qualitative description of all the measurement tools used in the included studies is provided in Table 2.

**Table 1:** Characteristics of included studies

Author name (year)	Study location	Student discipline	Age—Mean (SD) (unless otherwise specified)	Sex, n (%) female (unless otherwise specified)	Sample Size	Name of Measurement Tool (subcales)	Mean (SD) measurement score and/or % who have positive attitudes (author defined) towards patient centred care	Subscales score, mean (SD)
Ahmad et al. (2015)	Pakistan	Medicine	Not reported	557 (71.10%)	783	Patient-Practitioner Orientation Scale	3.40 (0.49)	Sharing: 3.18 (0.56); Caring: 3.63 (0.56)
Balentine et al. (2010)	U.S	Medicine	Not reported	70 (30%)	236	Patient-Practitioner Orientation Scale	4.50, no SD or CI provided	Not reported
Davis et al. (2006)	U.S	Medicine and Physician Assistant	Individual breakdown for each profession not reported. Entire sample mean age = 28.30	Individual breakdown for each profession not reported. Entire sample (32) = 15 males, 12 females, 5 unreported	Total 32, 14 students (6 medicine, 8 physician assistant)	Patient-Practitioner Orientation Scale	Medicine: 4.70 (0.30) Physician Assistant: 4.60 (0.30)	Not reported
Dockens et al. (2016)	U.S	Pre-service speech and hearing sciences	All: 22.60 (5.40) Low Exposure: 23.50 (7.50) Medium Exposure: 22.30 (4.30) High Exposure: 21.70 (2.80)	All: 75 (80.60) Low Exposure: 26 (78.80) Medium Exposure: 32 (82.10) High Exposure: 17 (81.00)	All: 93 Low Exposure: 33 Medium Exposure: 39 High Exposure: 21	Patient-Practitioner Orientation Scale	All: 4.13 (0.50) Low Exposure: 4.10 (0.40) Medium Exposure: 4.10 (0.50) High Exposure: 4.24 (0.50)	All: sharing: 4.29 (0.60), caring: 3.97 (0.40) Low Exposure: sharing 4.20 (0.60), caring: 3.95 (0.40) Medium Exposure: sharing 4.21 (0.50), 3.98 (0.40) High Exposure: sharing: 4.46 (0.70), 4.01 (0.40)
Fothan, Eshaq & Bakather (2019)	Saudi Arabia	Medicine	Not reported	75 (56.80%)	132	Patient-Practitioner Orientation Scale	4.00 (1.50)	sharing: 4.20 (1.50) caring: 3.80 (1.40)
Gaufberg et al. (2018)	U.S	Medicine	Not reported	Gold Humanism Honor Society: 52 (50.50%) Non-Gold Humanism Honor Society: 219 (45.40%)	All: 583 (103 Gold Humanism Honor Society, 480 non) at year 4: 570 (98 Gold Humanism Honor Society, 472 non) at year 3: 378 (64 Gold Humanism Honor Society, 314 non) at year 2: 389 (66 Gold Humanism Honor Society, 323 non) at year 1: 479 (80 Gold Humanism Honor Society, 399 non) Demographics table: (92 Gold Humanism Honor Society, 448 non-Gold Humanism Honor Society)	Patient-Practitioner Orientation Scale	Gold Humanism Honor Society: All: 4.45 (0.42) Year 1: 4.39 (0.46) Year 2: 4.44 (0.53) Year 3: 4.40 (0.47) Year 4: 4.49 (0.53) Non-Gold Humanism Honor Society: All: 4.27 (0.39) Year 1: 4.26 (0.43) Year 2: 4.33 (0.47) Year 3: 4.23 (0.52) Year 4: 4.28 (0.50)	Not reported
Grilo et al. (2013)	Portugal	Nursing	Male 1st year: 20.77(4.52) Male 2nd year: 22.68(3.95) Male 4th tear: 22.00(0.75) Male Total including nurses: 24.71(7.73) Female 1st year: 19.53(3.23) Female 2nd year: 20.82(2.44) Female 4th tear: 22.43(2.61) Female Total including nurses: 22.78(6.90)	1st: 207 (87.00) 2nd: 126 (80.80) 4th: 111 (85.40) Nurses: 84 (77.80) Total (including nurses): 528 (83.50)	1st year: 238 2nd year: 156 4th year: 130 Nursing students total: 524 nurses (ineligible: 108) study total: 632	Patient-Practitioner Orientation Scale	1st: 4.31 (0.40) 2nd: 4.70 (0.43) 4th: 4.96 (0.38) Nurses (exclude): 4.48 (0.53) Total (nurses included): 4.57 (0.49)	Sharing: 1st: 4.11 (0.55) 2nd: 4.62 (0.61) 4th: 4.94 (0.52) nurses(exclude): 4.25 (0.64) Total (nurses included): 4.43 (0.66) Caring: 1st: 4.51 (0.44) 2nd: 4.80 (0.44) 4th: 4.98 (0.40) nurses (exclude): 4.71 (0.60) total (nurses included): 4.71 (0.49)
Haidet et al. (2001)	U.S	Medicine	Not reported	120(41%)	293	Patient-Practitioner Orientation Scale	4.58 (0.46)	Not reported
Haidet et al. (2002)	U.S	Medicine	fourth year students: 25(2.30)	1st year: 118 (45) 3rd year: 65 (41) 4th year: 36 (41)	1st year: 263 3rd year: 158 4th year: 89 Total: 510	Patient-Practitioner Orientation Scale	Total: 4.57 (0.48) 1st: 4.61 3rd: 4.59 4th: 4.48	Not reported
Hammerich et al. (2019)	Canada, U.S., Wales, Denmark, France, Australia	Chiropractic	Age (% of sample) 17–19: 84 (5%) 20–24: 1060 (57%) 25–29: 523 (28%) 30–34: 95 (5%) 35 + : 87 (5%)	1048 (57%)	1858	Patient-Practitioner Orientation Scale	PPOS: Canadian Memorial Chiropractic College: 4.27 (0.46) Parker University: 4.06 (0.53) Northwestern Health Sciences University: 4.13 (0.46) Southern Denmark University: 4.11 (0.45) University of South Wales: 4.15 (0.43) Central Queensland University: 4.31 (0.54) L’Institut Franco-Europeen de Chiropraxie: 4.22 (0.43) Total: 4.18 (0.48)	Caring: Canadian Memorial Chiropractic College: 4.50 (0.48) Parker University: 4.25 (0.55) Northwestern Health Sciences University: 4.33 (0.48) Southern Denmark University: 4.55 (0.47) University of South Wales: 4.40 (0.49) Central Queensland University: 4.53 (0.54) L’Institut Franco-Europeen de Chiropraxie: 4.75 (0.46) Sharing: Canadian Memorial Chiropractic College: 4.05 (0.61) Parker University: 3.86 (0.68) Northwestern Health Sciences University: 3.94 (0.59) Southern Denmark University: 3.68 (0.62) Unoversity of South Wales: 3.91 (0.63) Central Queensland University: 4.09 (0.69) L’Institut Franco-European de Chiropraxie: 3.70
Henschen et al. (2015)	U.S	Medicine	Not reported	Traditional Curriculum: 30 (44%) Education- centred medical home: 40 (58%)	137 (Traditional curriculum = 68, education- centred medical home = 69)	Patient-Practitioner Orientation Scale	Traditional Curriculum: 4.3 (0.80) Education- centred Medical Home: 4.6 (0.50)	Not reported
Hirsh et al. (2012)	U.S	Medicine	Not reported	Not reported	67 (27 Cambridge integrated Clerkship, 40 traditional)	Patient-Practitioner Orientation Scale	Cambridge integrated clearkship: 5.00 Traditional: 4.87	Not reported
Hur, Cho & Choi (2017)	South Korea	Medicine	Not reported	75 (37.90%)	198 total (89 in 2006, 109 in 2009)	Patient-Practitioner Orientation Scale	3.90 (0.40)	Sharing: 3.61 (0.49) Caring: 4.18 (0.45)
Krupat et al. (2009)	U.S	Medicine	Not reported	Not reported	49 total (32 Principal clinical experience, 17 control)	Patient-Practitioner Orientation Scale	Principal clinical experience: 5.00 Control group: 4.90	Not reported
Lee et al. (2008)	Singapore	Medicine	Range 20–23	92 (40.70)	226	Patient-Practitioner Orientation Scale	4.10 (0.42)	Sharing: 3.84 (0.51) Caring: 4.36 (0.49)
Madham, Rajpurohit & Gayathri (2010)	India	Dentistry	26.18 (2.07)	90 (44.60%)	202	Patient-Practitioner Orientation Scale	3.38 (0.63)	Sharing: 3.11 (0.65) Caring: 3.5 (0.88)
Meirovich et al. (2016)	Israel	Medicine	22.9 (range 21–29)	25 (46%)	32 (16 experimental, 16 control)	Patient-Practitioner Orientation Scale	Experimental: 4.21 (0.37) Control: 4.26 (0.43)	Not reported
Michael, Dror & Miller (2019)	Israel	Medicine and Dentistry	27.49 (3.60)	359 (57.80%)	653	Patient-Practitioner Orientation Scale	4.45 (0.44)	Not reported
Moore (2009)	Nepal	Medicine	Not reported	12 (26.75)	63 total 45 students	Patient-Practitioner Orientation Scale	4.26	Not reported
Mudiyanse et al. (2015)	Sri Lanka	Medicine	23 (2.30)	289 (53.20%)	543 (254 males, 289 females)	Patient-Practitioner Orientation Scale	Male: 4.40 (0.60) Female: 4.40 (0.50)	Male Sharing: 3.90 (0.70) Caring: 4.80 (0.70) Female: Sharing: 4.00 (0.70) Caring: 4.90 (0.60)
Pers et al. (2019)	Poland	Medicine	Clinical Communication Course + : 23.49 (1.08) Clinical Communication Course-: 24.82 (0.85) Clinical Communication Course English + : 25.2 (2.03)	Clinical Communication Course + : 94 (60.26%) Clinical Communication Course-: 87 (73.73%) Clinical Communication Course English + : 20 (37.74%)	Clinical Communication Course + : 156 Clinical Communication Course-: 118 Clinical Communication Course English + : 53 Total: 327	Patient-Practitioner Orientation Scale	Clinical Communication Course + : 2.91 (0.50) Clinical Communication Course-: 2.74 (0.47)	Sharing Clinical Communication Course + (*n* = 160): 3.06 (0.63), range 1.44 -5.11 Clinical Communication Course- (*n* = 122): 2.95 (0.62), range 1.22–4.67 Caring: Clinical Communication Course + : 2.75 (0.51), range 1.11–4.11 Clinical Communication Course-: 2.52 (0.48), range 1.33–3.67
Ribeiro, Krupat & Amaral (2007)	Brazil	Medicine	Not reported	360 (48.8%)	738	Patient-Practitioner Orientation Scale	4.66 (0.44)	Caring Male: 5.04 (0.47) Caring Female: 5.26 (0.43) Sharing Male: 3.82 (0.58) Sharing Female: 4.18 (0.58) Total: Sharing: 4.10 (0.66) Caring: 5.20 (0.45)
Rosewilliam et al. (2019)	United Kingdom	Physiotherapy: 47 (22%) Medicine: 86 (41%) Nursing: 28 (13%) Speech and Language Therapy: 50 (24%)	22.7 (4.90)	176 (83%)	211	Patient-Practitioner Orientation Scale	18-item average, total score (SD of total score) 4.09, 73.62 (8.81)	9-item average, total score (SD of total score) Sharing: 4.30, 38.72 (5.4) Caring: 3.87, 34.91 (5.1)
Ross & Haidet (2011)	U.S	Physical Therapy	Not reported	Not reported	46	Patient-Practitioner Orientation Scale	Sum total mean (SD): 81.30 (7.70)	Sharing total mean(SD): 39.30 (4.70) Caring total mean: 41.90 (4.10)
Sweeney and Baker (2018)	United Kingdom	Medicine	Not reported	not reported	39	Patient-Practitioner Orientation Scale	78.8	Not reported
Tsimtsiou et al. (2007)	Greece	Medicine	Not reported	Year 4: 111 (46.20%) Year 6: 98 (40.30%)	Year 4: 240 students Year 6: 243 students The same cohort was surveyed twice	Patient-Practitioner Orientation Scale	Year 4: 3.96 Year 6: 3.81	Sharing Year 4: 3.50 Year 6: 3.24 Caring: Year 4: 4.41 Year 6 4.38
Harris et al. (2020)	Switzerland	Medicine	Not reported	195 (63.70)	306	Patient-Practitioner Orientation Scale- D12	4.19 (0.47)	Not reported
Liu et al. (2019)	China	Medicine	< = 22: 378 (73.70%) > 22 = 135 (26.30%)	Five year clinical category: 238 Seven-year: 72 Total: 310 (60.4%)	Total: 513 Five-year clinical category: 394 Seven-year clinical category 119	Chinese Revised-Patient-Practitioner Orientation Scale	3.63 (0.54)	Sharing: 2.88 (0.67) Caring: 4.53 (0.82)
McNair et al. (2016)	Australia	Medicine	Not reported	56% (likely 113, but unclear)	203	Adapted 9 questions from Patient-Practitioner Orientation Scale	Inner metropolitan: 4.00 (0.39) Outer metropolitan: 4.00 (0.36) Rural: 4.00 (0.35)	Not reported
El-Awaisi et al. (2017)	Qatar	Medicine: 6 Pharmacy: 24 Pharmacy technician: 6 Public health: 11	n (%) < 20: 4(8.59%) 20–24: 42(89.40%) 25–29: 1(2.10%)	44 (93.60%)	47	Readiness for Interprofessional Learning Scale	Median (IQR) 23 (5)	No relevant subscales
Hudson et al. (2016)	Australia	Medicine	Not reported	not reported	279	Readiness for interprofessional learning and attitude to patient-centredness survey	Mean = 23.42, SEM = 0.11	No relevant subscales
Norris et al. (2015)	U.S	Medicine, Pharmacy, Nursing, Public Health	Exploratory Factor Analysis: 13–22: 14 23–32: 230 33–42: 65 43–52:21 53–62: 10 63–72: 2 Confirmatory Factor Analysis: 13–22: 7 23–32: 239 33–42: 54 43–52: 23 53–62: 12 63–72: 0 Total: 13–22: 21(3.10) 23–32: 469 (69.30) 33–42: 119 (17.60) 43–52: 44 (6.50) 53–62: 22 (3.20) 63–72: 2 (0.30)	Exploratory Factor Analysis: 208 (60.80) Confirmatory Factor Analysis: 202 (60.30) Total: 410 (60.60)	Exploratory Factor Analysis: 342 Confirmatory Factor Analysis: 336 Total: 678	Readiness for Interprofessional Learning Scale	Exploratory Factor Analysis: 4.60 (0.50) Confirmatory Factor Analysis: 4.62 (0.46)	No relevant subscales
Zaudke et al. (2016)	U.S	Medicine, Nursing, Pharmacy	Not reported	Not reported	252 Medicine: 153 Nursing: 23 Pharmacy: 46	Readiness for Interprofessional Learning Scale	4.65 (0.47)	No relevant subscales
Zeeni et al. (2016)	Lebanon	Nursing, Nutrition, Pharmacy, Social Work and Medicine	21.1 (0.12)	108 (70.10%)	157 (46 medicine, 67 pharmacy, 21 nursing, 23 nutrition)	Readiness for Interprofessional Learning Scale	22.75 (2.46)	No relevant subscales
Batenburg (1997)	Netherlands	Medicine	Not reported	Not reported	476	Doctor-Patient Scale	3.50 (0.24)	Not reported
Batenburg et al. (1999)	Netherlands	Medicine	Not reported	24 (60%)	40	Doctor-Patient Scale	General Practice Clerks: 64.0% (5.60), Surgery Clerks 58.6% (6.30)	Not reported
Bombeke et al. (2011)	Belgium	Medicine	Communication Skills Training* (-): 24.80, Communication Skills Training ( +): 24.60	Communication Skills Training (-): 30 (63%), Communication Skills Training ( +): 22 (59%)	85 (Communication Skills Training (-): 48, Communication Skills Training ( +): 37)	Doctor-Patient Scale	Communication Skills Training (-): 3.45 (0.30) Communication Skills Training ( +): 3.54 (0.22)	Not reported
Noble et al. (2007)	United Kingdom	Medicine	19 (2.00), range 17 -31	270 (59%)	454	Doctor-Patient Scale	Old Curriculum (*n* = 199): 3.20 (0.20) New curriculum (*n* = 255): 3.22 (0.20)	No relevant subscales
Davis et al. (2018)	U.S	Nursing and Medical Assistant	Bachelor of Science Nursing: 26.80 Medical Assistant: 30.80	BSN: 10 (90.90) MA: 18 (85.70)	Bachelors of Science Nursing: 11 Medical Assistant: 21	Interprofessional attitudes scale	Entire sample size pooled (*n* = 32): 33.60 (2.40)	No relevant subscales
King & Violato (2020)	Canada	Nutrition, Dental Hygiene, Dentistry, Medical Laboratory Science, Medicine, Radiation Therapy, Nursing, Pharmacy and Pharmaceutical Sciences, Kinesiology, Physical Therapy, Occupational Therapy, Speech Language Pathology	Not reported	Not Reported	337	Interprofessional Attitude Scale	Patient centredness for all participants: 6.65 (0.49)	No relevant subscales
Hardeman et al. (2015)	U.S	Medicine	Race: age: n(%) White: 18–24: 2085 (72%) 25- 35 or older: 811 (28%) African America: 18–24: 195 (65%) 25–35 or older: 104 (35%)	Race: Female: n (%) White: Female: 1390 (48%) African American: Female: 198 (66%)	White: 2890 African American: 301 Total: 3191	Health Beliefs Attitudes Survey	Low Health Beliefs Attitudes Survey: 1543 High Health Beliefs Attitudes Survey: 1667	Not reported
Burnard & Morrison (1991)	not reported	District nursing, Health visiting, Nursing	Not reported	Not reported	District nursing student: 24 health visiting students: 24: Nursing students: 21	Nelson-Jones and Patterson Counsellor Attitude Scale	Mean score (range) District Nursing: 37 (24–47) Health Visiting: 45 (32–59) Nursing: 44 (24–63)	Not reported
Rolfe (1994)	United Kingdom	Nursing	18–22: 189 (3.81) 23–27: 60 (5.08) 28–32: 19 (5.82) 33 + : 45 (4.51)	Not reported	315	Patient-centredness Multi-choice Questionnaire	37.78 (4.41)	Not reported
Mirsu-Pau, Tucker & Hardt (2012)	U.S	Medicine	26 (3.40), range 22–56	114 (53%)	216	Tucker-Culturally Sensitive Health Care Inventory Provider Form	3.30 (0.37)	Not reported
Beach et al. (2007)	U.S	Medicine	21–30: 146 (86%), > 30: 22 (14%)	76 (45%)	177	No name reported	Cut point of "patient centredness" created at a score of 68. 85 scored < 68, 83 scored > 68	No relevant subscales
Hauer et al. (2010)	U.S	Medicine	Not reported	169 (54%)	336	No name reported	75 (6.60) range: 53–100	Not reported
Ster et al. (2015)	Slovenia	Medicine	Not reported	109 (68.60%)	159	No name reported	4.57 (1.44)	No relevant subscales
Stoner et al. (2018)	U.S	Osteopathic Medicine	Not reported	Not reported	69	No name reported	3.39 (0.35)	No relevant subscales
Welch Bacon et al. (2018)	U.S	Athletic training	23.29 (2.05)	138 (62.44%)	221	No name reported	Patient Centred Care implementation: 3.20 (0.38) Rating of importance of Patient Centred Care: 3.61 (0.35)	No relevant subscales

*U.S.* United States

**Table 2:** Measurement tools and their subscales identified in the included studies

Name of tool	Construct	N
Patient Practitioner Orientation Scale (PPOS)	The scale contains 18 items scored on a 6-point Likert scale (1 = strongly disagree to 6 = strongly agree) where higher scores indicate higher attitudes towards patient centred care. The scale consists of two subscales (sharing and caring) each with 9 questions. The overall score is computed as the mean of the scores for the 18 items. Sharing and caring scores are computed as the mean of the score for their respective scales	26
Adapted-Patient Practitioner Orientation Scale	The scale contains 9 items scored on a 5-point Likert scale (1 = strongly disagree to 5 = strongly agree) where higher scores indicate more attitudes towards patient centred care	1
CR-Patient Practitioner Orientation Scale	The scale contains 11 items scared on a 5-point Likert scale (1 = strongly disagree to 5 = strongly agree) where higher scores indicate more attitudes towards patient centred care	1
Patient Practitioner Orientation Scale- D12	The scale contains 12 items scored on a 6-point Likert scale (1 = strongly disagree to 6 = strongly agree) where higher scores indicate higher attitudes towards patient centred care. The scale consists of two subscales (sharing and caring) each with 6 questions. The overall score is computed as the mean of the scores for the 12 items. Sharing and caring scores are computed as the mean of the score for their respective scales	1
Readiness for Interprofessional Learnnig Scale (RIPLS)	The scale contains 26 items, 5 of which assess attitudes towards patient centred care scored on a 5-point Likert scale (1 = strongly disagree to 5 = strongly agree) where higher scores indicate more attitudes towards patient centred care	5
Doctor-Patient Scale	The scale contains 48 items scored on a 5-point Likert scale (1 = strongly disagree to 5 = strongly agree) where higher scores indicate more attitudes towards patient-centred care	4
Interprofessional attitudes scale (IPAS)	The scale contains 27 items scored on a 7-point Likert scale (1 = strongly disagree to 7 = strongly agree) where higher scores indicate more attitudes towards patient centred care	2
Patient-centredness Multi-choice Questionnaire (PMQX)	The scale contains 10 items. The details of the scoring were not reported	1
Health Beliefs Attitudes Survey (HBAS)	The scale contains 15 items scored on a 6-point Likert scale (1 = strongly disagree to 5 = strongly agree) where higher scores indicate more attitudes towards patient centred care	1
Nelson-Jones and Patterson Counsellor Attitude Scale	The scale contains 70 items. Participants are asked to read each item and to respond by indicating that they agreed with, disagreed with or could not decide about each item	1
Tucker-Culturally Sensitive Health Care Inventory Provider Form (T-CSHCI)	The scale contains 53 items of which 23 items assesses attitudes towards patient centred care scored on a 4-point Likert scale (1 = strongly disagree to 4 = strongly agree) where higher scores indicate more attitudes towards patient-centred care	1
No name reported (Beach et al. 2007)	The scale contains 9 items scored on a 5-point Likert scale (1 = strongly disagree to 5 = strongly agree) where higher scores indicate more attitudes towards patient centred care	1
No name reported (Ster et al. 2015)	The scale contains 60 items scored of which 1 item assesses attitudes towards patient centred care scored on a 7-point Likert scale (1 = total disagreement to 7 = total agreement) where a higher score indicates more attitudes towards patient centred care	1
No name reported (Stoner et al. 2018)	The scale contains 22 items of which 9 assesses attitudes towards patient centred care scored on 5-point Likert scale (1 = strongly disagree to 5 = strongly agree) where higher scores indicates more attitudes towards patient centred care	1
No name reported (Hauer 2010 et al.)	The scale contains 9 items scored on a 5-point Likert scale (1 = strongly disagree to 5 = strongly agree) where higher scores indicate more attitudes towards patient centred care	1
No name reported (Welch Bacon 2018 et al.)	The scale contains 71 items of which 11 assesses attitudes towards patient centred care scored on a 4-point Likert scale (1 = strongly disagree to 4 = strongly agree) where higher scores indicate more attitudes towards patient centred care	1

*N* Number of studies using the tool

Mean methodological quality assessed using a modified 10-item Downs and Black checklist was 9.04 (95% Confidence Interval [CI]: 8.73, 9.35; minimum 6; maximum 10). The most commonly omitted methodological quality indicators were a lack of appropriate reporting of probability values, subjects not being representative of the entire population from which they were recruited, and participants not being representative of the population from which they were recruited. A comprehensive breakdown of the methodological quality for each study is provided in Appendix 4.

### Healthcare students’ attitudes towards patient centred care

**Fig. 2:**
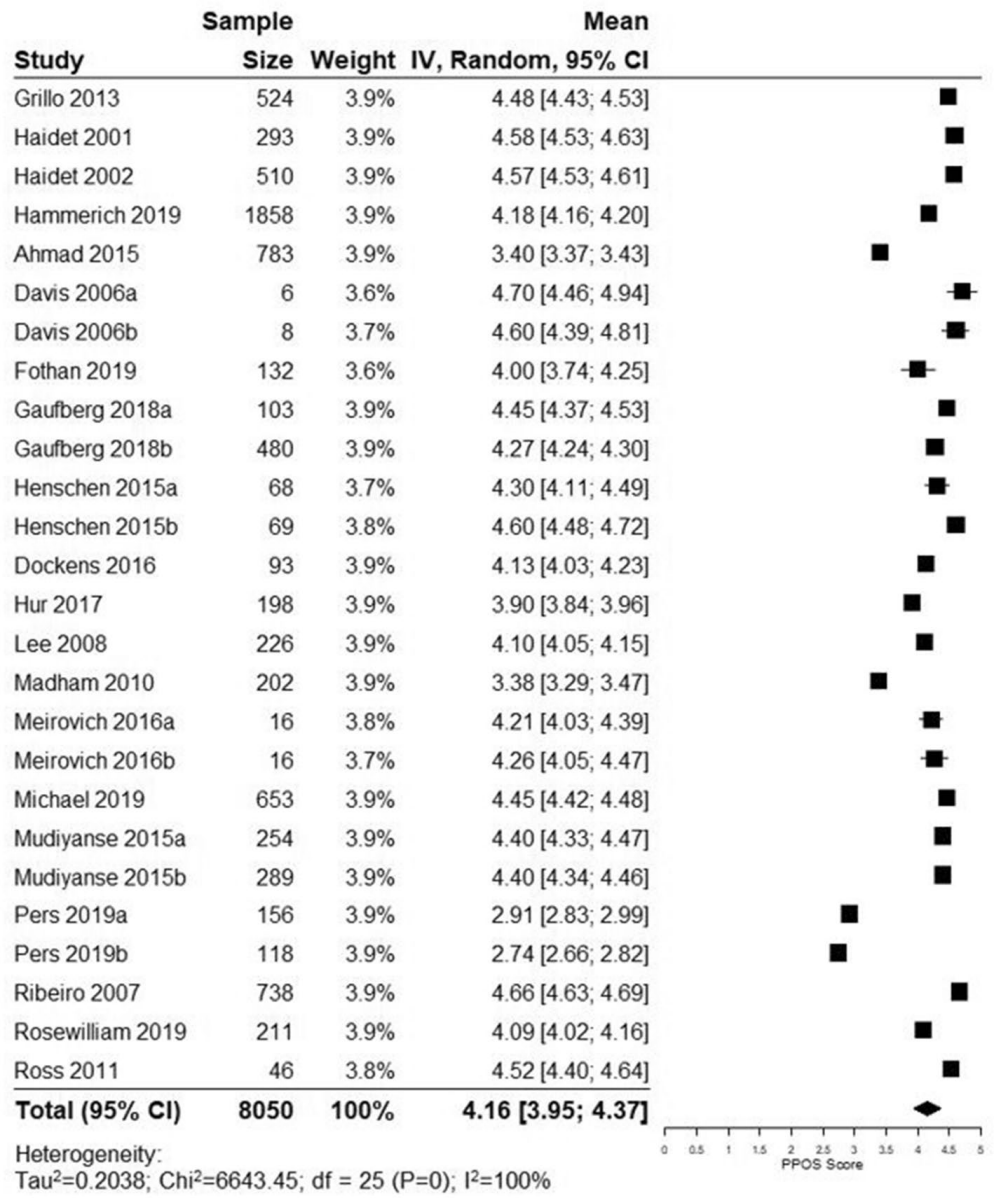
Forest plot of mean pooled PPOS score and 95% CI for healthcare students

Due to limited data, we were only able to perform a meta-analysis of studies that used the PPOS (0–6 scale) to assess healthcare students’ attitudes towards patient centred care. There were 20 studies with 26 total groups included in the meta-analysis (*n* = 8050). Most studies analyzed medical students (*n* = 18) followed by a mix of healthcare students (*n* = 2), nursing (*n* = 1), physician assistant (*n* = 1), dentistry (*n* = 1), speech therapy (*n* = 1), chiropractic (*n* = 1), and physical therapy (*n* = 1) students. Overall, the pooled mean score on the PPOS was 4.16 (95% CI: 3.95, 4.37; I^2^: 100%) (Fig. 2).

### Factors influence on attitudes towards patient centred care

**Fig. 3:**
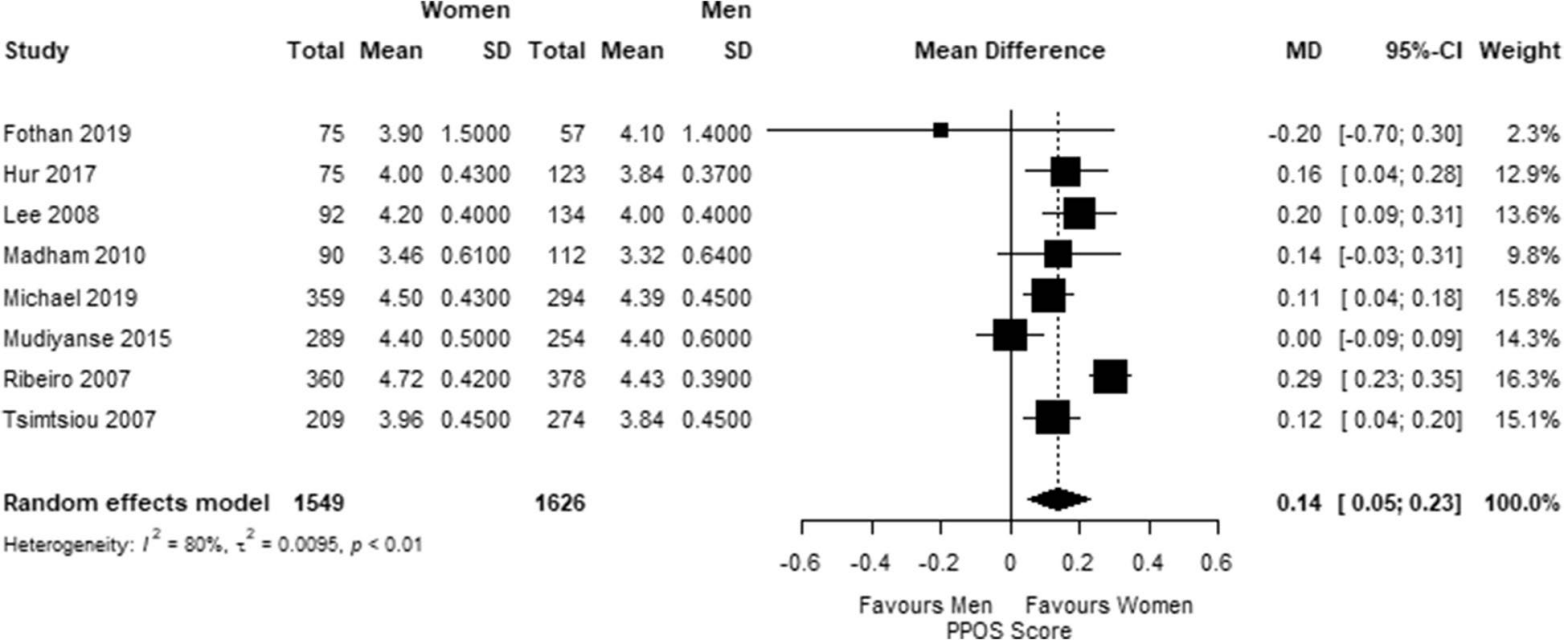
Forest plot of mean PPOS score and 95% CI difference between female and male healthcare students

**Fig. 4:**
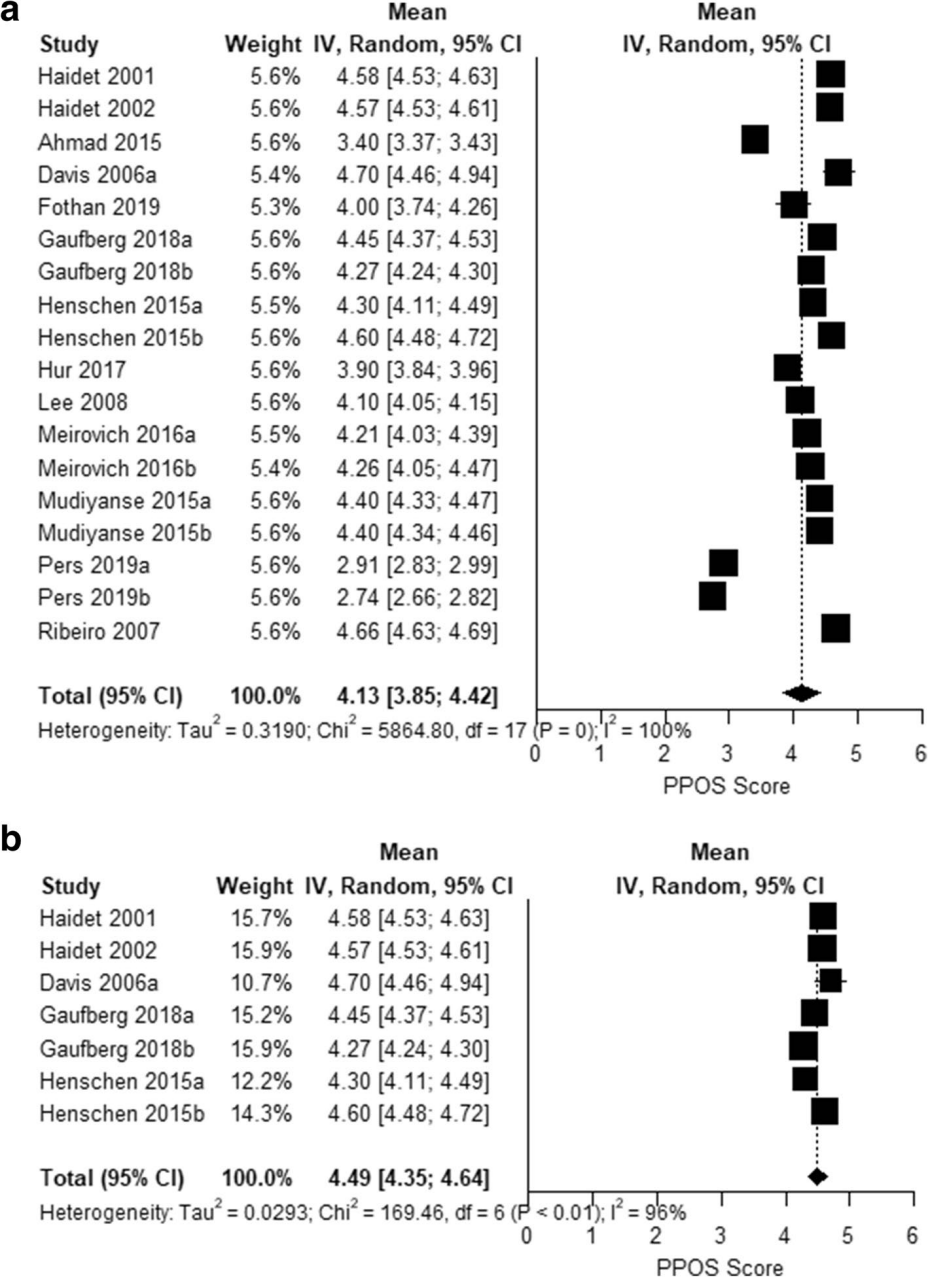
**a-b** Forest plots of mean PPOS score and 95% CI for medical students only

Sex, profession, and geographical location were the only factors with data available to conduct analyses to address our secondary aim of potential influence on healthcare students’ attitudes towards patient centred care. Three analyses (sex, only medical students, and only medical students in the U.S.) were conducted attempting to explain heterogeneity. Eight studies reported PPOS data stratified by sex. Among these, there were 3175 total healthcare students included (1626 men and 1549 women). The total PPOS mean score was slightly higher in women (MD 0.14, 95% CI: 0.05, 0.23; I^2^: 80%, *n* = 8 studies) (Fig. 3). PPOS mean scores were similar among subgroups of only medical students with a pooled mean score of 4.13 (95% CI: 3.85, 4.42; I^2^: 100%, *n* = 13 studies with 18 total groups) (Fig. 4a) and only U.S. medical students with a mean score of 4.49 (95% CI: 4.35, 4.64; I^2^: 95%, *n* = 5 studies with 7 total groups) (Fig. 4b). Hence, none of the analyses was able to substantially explain the heterogeneity found in the meta-analysis.

## Discussion

This is the first systematic review to summarize the measurement tools used to assess healthcare students’ attitudes towards patient centred care and quantify their attitudes. There were 16 measurement tools used to assess attitudes towards patient centred care across 49 included studies with the most common being the PPOS. Women have slightly higher attitudes towards patient centred care compared to men, and medical students, particularly those from the U.S., have slightly higher attitudes towards patient centred care than healthcare students’ overall.

Patient centred care is consistently recommended in clinical practice guidelines for a variety of conditions (e.g., musculoskeletal pain, depression, end of life care etc.) [77–79]. Additionally, research suggests that patient centred care is associated with higher patient satisfaction [80, 81], improved patient outcomes [3, 4, 82], and lower healthcare costs [83, 84]. Unfortunately, our findings indicate that students have low attitudes towards patient centred care overall, according to the classification by Krupat et al. [80]. Mean PPOS scores should be interpreted as high (mean score > 5.00; patient centred), medium (mean score 4.57–4.99), or low (mean score < 4.57, doctor centred). Meta-analysis of studies in our review reported a total mean score of 4.16 on the PPOS. Our findings are similar to a recent systematic review that included four studies measuring physicians’ attitudes towards patient centred care using the PPOS [12]. Those four studies reported total mean PPOS scores of 3.98, 4.08, 4.55, and 4.97 [5, 81, 85, 86]. The PPOS has demonstrated acceptable validity and adequate reliability among healthcare students [70, 87, 88]. While healthcare students are learning new information during their education and have limited time to focus on other aspects of patient care, the results of our review and Pollard et al*.* [12] indicate that both healthcare students and professionals have low attitudes towards patient centred care. Due to the known positive effect of patient centred care on healthcare outcomes and costs, it is important to develop and test strategies to improve healthcare students’ and professionals’ attitudes towards and implementation of patient centred care.

We found that female healthcare students have higher attitudes towards patient centred compared to males, which is similar to previous studies [70]. However, the difference between males and females was small and both groups would still be classified as having low attitudes towards patient centred care. These results imply that healthcare students require training to improve attitudes towards patient centred care and special considerations may be required for male students, but the importance of the observed difference between males and females is not clear. Analysis of only medical students found similar mean PPOS score as overall healthcare students indicating that attitudes may not differ widely between healthcare professions. However, medical students from the U.S. reported higher attitudes towards patient centred care compared to healthcare students’ overall, but again, the pooled mean score of the attitudes were still considered low towards patient centred care [6, 25, 29, 31, 36]. These results imply that there may be cultural or societal differences that may influence attitudes towards patient centred care.

Future studies assessing healthcare students’ attitudes towards patient centred care should use the PPOS to allow for comparability to previous literature or aim to validate existing tools. Many studies (41%) included in our systematic review used tools that have not demonstrated validity and reliability or have been used only once, making it difficult to interpret and compare the results of studies. Studies using a different measurement tool should look to validate and compare the psychometric properties with the PPOS.

There were only self-reported measurement tools (e.g., PPOS, RIPLS, Doctor-Patient Scale, IPAS etc.) found in our review therefore, there may be a need for objective tools used to measure patient centred care. Longitudinal studies are also needed to assess whether healthcare students’ attitudes persist into clinical practice or if attitudes evolve throughout training and with years of clinical experience. Additionally, future studies should evaluate if healthcare education can positively influence and increase healthcare students’ attitudes towards patient centred care.

Our systematic review has some limitations. We found high heterogeneity in our main meta-analyses, and this could not be explained with analyses of available factors that may influence attitudes towards patient centred care. We only included studies in English, so it is possible important data from non-English articles was missed. Additionally, our electronic database search was not conducted in all available databases, such as the Education Resources Information Center (ERIC) database or grey literature, manual searching of educational journals was not conducted, nor was pursuing the publications of relevant scholars and authors was conducted. Therefore, it is possible that relevant studies were not captured. However, our search strategy was tested independently by two research librarians, reference list screening was performed, and since all studies were related to healthcare students, it is likely they would be indexed in medical and healthcare databases. Therefore, it is unlikely that relevant literature was not included. The results of medical students and U.S. medical students only should be interpretated with caution since the majority of included studies were conducted in the U.S. therefore the results may not represent non-U.S. healthcare students.

## Conclusions

We identified 16 different measurement tools that have been used to assess healthcare students’ attitudes towards patient centred care, with the most popular being the PPOS. Our results suggest that healthcare students have low attitudes towards patient centred care when measured by the PPOS. There is considerable opportunity to increase healthcare students’ attitudes toward patient centred care in order to improve patient outcomes and decrease healthcare costs. Universities have a unique opportunity to shape their curriculum to emphasize features of patient centred care. Specific classes to practice, role-play, and discuss ways to increase the dimensions of patient centred care (e.g., biopsychosocial perspective, the patient as a person, sharing power and responsibility,therapeutic alliance, and doctor as a person) may allow for increased attitudes towards patient centred care by healthcare students. This increase in attitude towards patient centred care and the dimensions that encompass it may lead to a better patient-doctor relationship that has previously led to decrease healthcare costs.

## Supplementary Information

**Additional file 1:** **Appendix 1.** PRISMA 2020 Table. **Appendix 2.** Medline, CINAHL, andEmbase search strategy. **Appendix 3.** Modified Downs andBlack checklist. **Appendix 4.** Risk of Bias table.

## Data Availability

The authors included all data relevant to the study in the manuscript or appendix.

## Abbreviations

PPOS: Patient-Practitioner Orientation Scale
PRISMA: Preferred Reporting Items for Systematic Reviews and Meta-Analysis Protocols
SD: Standard deviation
IQR: Median interquartile range
CI: Confidence interval
U.S.: United States
RIPLS: Readiness for Interprofessional Learning Scale
IPAS: Interprofessional Attitudes Scale
