# Supplementary material for: Healthcare students’ attitudes towards patient centred care: a systematic review with meta-analysis
Source: BMC Med Educ. 2022 Apr 27;22:324. doi: 10.1186/s12909-022-03371-1 (PMC9047330; doi:10.1186/s12909-022-03371-1)
Supplement: Supplementary file 1 — Additional file 1: Appendix 1. PRISMA 2020 Table. Appendix 2. Medline, CINAHL, andEmbase search strategy. Appendix 3. Modified Downs andBlack checklist. Appendix 4. Risk of Bias table. [file 12909_2022_3371_MOESM1_ESM.docx]

**Appendix Legend**

Appendix 1: PRISMA 2020 Table

Appendix 2: Medline, CINAHL, and Embase search strategy

Appendix 3: Modified Downs and Black checklist

Appendix 4: Risk of Bias table

Appendix 1

| **Section and Topic** | **Item #** | **Checklist item** | **Location where item is reported** |
| --- | --- | --- | --- |
| **TITLE** | | |  |
| Title | 1 | Identify the report as a systematic review. | Line number 2 |
| **ABSTRACT** | | |  |
| Abstract | 2 | See the PRISMA 2020 for Abstracts checklist. | Page 2 |
| **INTRODUCTION** | | |  |
| Rationale | 3 | Describe the rationale for the review in the context of existing knowledge. | Line number 84-90 |
| Objectives | 4 | Provide an explicit statement of the objective(s) or question(s) the review addresses. | Line number 93-90 |
| **METHODS** | | |  |
| Eligibility criteria | 5 | Specify the inclusion and exclusion criteria for the review and how studies were grouped for the syntheses. | Line number 113-117 |
| Information sources | 6 | Specify all databases, registers, websites, organisations, reference lists and other sources searched or consulted to identify studies. Specify the date when each source was last searched or consulted. | Line number 103-111 |
| Search strategy | 7 | Present the full search strategies for all databases, registers and websites, including any filters and limits used. | Appendix 1 |
| Selection process | 8 | Specify the methods used to decide whether a study met the inclusion criteria of the review, including how many reviewers screened each record and each report retrieved, whether they worked independently, and if applicable, details of automation tools used in the process. | Line number 118-123 |
| Data collection process | 9 | Specify the methods used to collect data from reports, including how many reviewers collected data from each report, whether they worked independently, any processes for obtaining or confirming data from study investigators, and if applicable, details of automation tools used in the process. | Line number 125-136 |
| Data items | 10a | List and define all outcomes for which data were sought. Specify whether all results that were compatible with each outcome domain in each study were sought (e.g. for all measures, time points, analyses), and if not, the methods used to decide which results to collect. | Line number 126-136 |
|  | 10b | List and define all other variables for which data were sought (e.g. participant and intervention characteristics, funding sources). Describe any assumptions made about any missing or unclear information. | Line 126-136 |
| Study risk of bias assessment | 11 | Specify the methods used to assess risk of bias in the included studies, including details of the tool(s) used, how many reviewers assessed each study and whether they worked independently, and if applicable, details of automation tools used in the process. | Line number 137-147 |
| Effect measures | 12 | Specify for each outcome the effect measure(s) (e.g. risk ratio, mean difference) used in the synthesis or presentation of results. | Line number 149-154 |
| Synthesis methods | 13a | Describe the processes used to decide which studies were eligible for each synthesis (e.g. tabulating the study intervention characteristics and comparing against the planned groups for each synthesis (item #5)). | Line number 113-117 |
|  | 13b | Describe any methods required to prepare the data for presentation or synthesis, such as handling of missing summary statistics, or data conversions. | Line number 151-157 |
|  | 13c | Describe any methods used to tabulate or visually display results of individual studies and syntheses. | Line number 149-150 |
|  | 13d | Describe any methods used to synthesize results and provide a rationale for the choice(s). If meta-analysis was performed, describe the model(s), method(s) to identify the presence and extent of statistical heterogeneity, and software package(s) used. | Line number 151-157 |
|  | 13e | Describe any methods used to explore possible causes of heterogeneity among study results (e.g. subgroup analysis, meta-regression). | Line number 155 |
|  | 13f | Describe any sensitivity analyses conducted to assess robustness of the synthesized results. | N/A |
| Reporting bias assessment | 14 | Describe any methods used to assess risk of bias due to missing results in a synthesis (arising from reporting biases). | Line number 138-147 |
| Certainty assessment | 15 | Describe any methods used to assess certainty (or confidence) in the body of evidence for an outcome. | N/A |
| **RESULTS** | | |  |
| Study selection | 16a | Describe the results of the search and selection process, from the number of records identified in the search to the number of studies included in the review, ideally using a flow diagram. | Line number 159-167 and Figure 1 |
|  | 16b | Cite studies that might appear to meet the inclusion criteria, but which were excluded, and explain why they were excluded. | Line number 166-167 |
| Study characteristics | 17 | Cite each included study and present its characteristics. | Line number 159; 168-176 |
| Risk of bias in studies | 18 | Present assessments of risk of bias for each included study. | Line number 177-183 and appendix 3 |
| Results of individual studies | 19 | For all outcomes, present, for each study: (a) summary statistics for each group (where appropriate) and (b) an effect estimate and its precision (e.g. confidence/credible interval), ideally using structured tables or plots. | Table 1 and figure 1 |
| Results of syntheses | 20a | For each synthesis, briefly summarise the characteristics and risk of bias among contributing studies. | Line number 187-207 |
|  | 20b | Present results of all statistical syntheses conducted. If meta-analysis was done, present for each the summary estimate and its precision (e.g. confidence/credible interval) and measures of statistical heterogeneity. If comparing groups, describe the direction of the effect. | Line number 187-207 |
|  | 20c | Present results of all investigations of possible causes of heterogeneity among study results. | Line number 187-207 |
|  | 20d | Present results of all sensitivity analyses conducted to assess the robustness of the synthesized results. | N/A |
| Reporting biases | 21 | Present assessments of risk of bias due to missing results (arising from reporting biases) for each synthesis assessed. | Line number 177-183 and appendix 3 |
| Certainty of evidence | 22 | Present assessments of certainty (or confidence) in the body of evidence for each outcome assessed. | N/A |
| **DISCUSSION** | | |  |
| Discussion | 23a | Provide a general interpretation of the results in the context of other evidence. | Line number 208-227 |
|  | 23b | Discuss any limitations of the evidence included in the review. | Line number 258-263 |
|  | 23c | Discuss any limitations of the review processes used. | Line number 258-263 |
|  | 23d | Discuss implications of the results for practice, policy, and future research. | Line number 248-256 |
| **OTHER INFORMATION** | | |  |
| Registration and protocol | 24a | Provide registration information for the review, including register name and registration number, or state that the review was not registered. | Line number 101 |
|  | 24b | Indicate where the review protocol can be accessed, or state that a protocol was not prepared. | Line number 101 |
|  | 24c | Describe and explain any amendments to information provided at registration or in the protocol. | N/A |
| Support | 25 | Describe sources of financial or non-financial support for the review, and the role of the funders or sponsors in the review. | Line number 282 |
| Competing interests | 26 | Declare any competing interests of review authors. | Line number 279 |
| Availability of data, code and other materials | 27 | Report which of the following are publicly available and where they can be found: template data collection forms; data extracted from included studies; data used for all analyses; analytic code; any other materials used in the review. | Line number 277 |

*From:*  Page MJ, McKenzie JE, Bossuyt PM, Boutron I, Hoffmann TC, Mulrow CD, et al. The PRISMA 2020 statement: an updated guideline for reporting systematic reviews. BMJ 2021;372:n71. doi: 10.1136/bmj.n71

Appendix 2

*Medline (OVID) Search Strategy*

1. exp Patient-Centered Care/
2. Patient Care Planning/
3. Patient Participation/
4. Decision Making, Shared/
5. (patient* adj3 (tailor* or centered* or centred* or centric* or focus* or oriented*)).mp.
6. (person* adj3 (tailor* or centered or centred* or centric* or focus* or oriented*)).mp.
7. (client* adj3 (tailor* or centered* or centred* or centric* or focus* or oriented*)).mp.
8. (people* adj3 (tailor* or centered* or centred* or centric* or focus* or oriented*)).mp.
9. (patient* adj3 (empower* or engag* or activat* or participat* or perspectiv*)).mp.
10. patient care planning.mp.
11. ((share* or sharing) adj3 decision*).mp.
12. OR/ 1-11 [ *** patient-centred care ]
13. exp Attitude/
14. exp "Attitude of Health Personnel"/
15. exp Health Knowledge, Attitudes, Practice/
16. Empathy/
17. Perception/
18. (attitud* or empath* or perception* or perceiv* or perspectiv* or view*).mp.
19. OR/ 13-18 [ *** attitudes ]
20. Education, Medical/
21. Education, Medical, Undergraduate/
22. exp Education, Medical, Graduate/
23. exp Students, Health Occupations/
24. Students, Medical/
25. Students, Nursing/
26. Students, Pharmacy/
27. Students, Dental/
28. (student* or undergraduate* or graduate* or intern or interns or learner*).mp.
29. OR/ 20-28 [ *** healthcare students ]
30. "Surveys and Questionnaires"/
31. (measure* adj3 tool*).mp.
32. (questionnaire* or survey* or instrument* or assessment* or scale* or measure*).mp.
33. Patient-Practitioner Orientation Scale.mp.
34. OR/ 30-33 [ *** surveys/tools ]
35. 12 and 19 and 29 and 34
36. (comment or clinical conference or congress or consensus development conference or editorial or letter or guideline or practice guideline or case reports).pt.
37. 35 not 36

*CINAHL Plus (EBSCO) Search Strategy*

1. (MH "Patient Centered Care")
2. (MH "Consumer Participation")
3. (MH "Decision Making, Shared")
4. (MH "Patient Care Plans")
5. TI (patient* n3 (tailor* or centered* or centred* or centric* or focus* or oriented*)) or AB (patient* n3 (tailor* or centered* or centred* or centric* or focus* or oriented*))
6. TI (person* n3 (tailor* or centered* or centred* or centric* or focus* or oriented*)) or AB (patient* n3 (tailor* or centered* or centred* or centric* or focus* or oriented*))
7. TI (client* n3 (tailor* or centered* or centred* or centric* or focus* or oriented*)) or AB (client* n3 (tailor* or centered* or centred* or centric* or focus* or oriented*))
8. TI (people* n3 (tailor* or centered* or centred* or centric* or focus* or oriented*)) or AB (people* n3 (tailor* or centered* or centred* or centric* or focus* or oriented*))
9. TI (patient* n3 (empower* or engag* or activat* or participat* or perspectiv*)) or AB (patient* n3 (empower* or engag* or activat* or participat* or perspectiv*))
10. TI (consumer* n3 (empower* or engag* or activat* or participat* or perspectiv*)) or AB (consumer* n3 (empower* or engag* or activat* or participat* or perspectiv*))
11. TI patient care n3 plan* or AB patient care n3 plan*
12. TI (share* or sharing) n3 decision* or AB (share* or sharing) n3 decision*
13. OR / 1-12 [*** patient-centred care ]
14. (MH "Attitude+")
15. (MH "Attitude of Health Personnel")
16. (MH "Empathy")
17. (MH "Health Knowledge")
18. (MH "Perception")
19. TI (attitud* or empath* or perception* or perceiv* or perspectiv* or view*) or AB (attitud* or empath* or perception* or perceiv* or perspectiv* or view*)
20. OR/ 14-19 [ *** attitudes ]
21. (MH "Education, Medical+")
22. (MH "Education, Health Sciences+")
23. (MH "Students, Health Occupations+")
24. (MH "Students, Medical")
25. (MH "Students, Nursing")
26. (MH "Students, Dental")
27. (MH "Students, Pharmacy")
28. TI (student* or undergraduate* or graduate* or intern or interns or learner*) or AB student* or undergraduate* or graduate* or intern or interns or learner*)
29. OR/ 21-28 [*** healthcare students ]
30. (MH "Surveys")
31. (MH "Questionnaires+")
32. TI (measure* n3 tool*) or AB (measure* n3 tool*)
33. TI (questionnaire* or survey* or instrument* or assessment* or scale* or measure*) or AB (questionnaire* or survey* or instrument* or assessment* or scale* or measure*)
34. TI Patient-Practitioner Orientation Scale or AB Patient-Practitioner Orientation Scale
35. OR/ 30-34 [ *** surveys/ tools ]
36. 13 AND 20 AND 29 AND 35
37. 36 NOT PT (abstract or brief item or case study or commentary or doctoral dissertation or editorial or letter or practice guidelines or proceedings)

*Embase (OVID) Search Strategy*

1. patient participation/
2. patient care planning/
3. shared decision making/
4. (patient* adj3 (tailor* or centered* or centred* or centric* or focus* or oriented*)).mp.
5. (person* adj3 (tailor* or centered or centred* or centric* or focus* or oriented*)).mp.
6. (client* adj3 (tailor* or centered* or centred* or centric* or focus* or oriented*)).mp.
7. (people* adj3 (tailor* or centered* or centred* or centric* or focus* or oriented*)).mp.
8. (patient* adj3 (empower* or engag* or activat* or participat* or perspectiv*)).mp.
9. patient care planning.mp.
10. ((share* or sharing) adj3 decision*).mp.
11. OR/ 1-10 [ *** patient-centred care ]
12. exp attitude/
13. exp health personnel attitude/
14. attitude to health/
15. empathy/
16. perception/
17. (attitud* or empath* or perception* or perceiv* or perspectiv* or view*).mp.
18. OR/ 12-17 [ *** attitudes/perceptions ]
19. exp medical education/
20. undergraduate student/
21. graduate student/
22. exp medical student/
23. exp nursing student/
24. exp pharmacy student/
25. exp dental student/
26. (student* or undergraduate* or graduate* or intern or interns or learner*).mp.
27. OR/ 19-26 [ *** healthcare students ]
28. exp health care survey/
29. health survey/
30. exp questionnaire/
31. (measure* adj3 tool*).mp.
32. (questionnaire* or survey* or instrument* or assessment* or scale* or measure*).mp.
33. Patient-Practitioner Orientation Scale.mp.
34. OR/ 28-33 [ *** surveys/tools ]
35. 11 and 18 and 27 and 34
36. limit 35 to exclude medline journals
37. (books or chapter or conference abstract or conference paper or conference review or editorial or letter).pt.
38. 36 not 37

Appendix 3

10 item modified ‘Downs and Black’ checklist

Modified ‘Downs and Black’ checklist including descriptors

| Checklist item | Scoring system |
| --- | --- |
| 1. Is the hypothesis/aim/objective of the study clearly described? | Yes or no (1,0) |
| 1. Are the main outcomes to be measured clearly described in the introduction or methods section?  - If the main outcomes are first mentioned in the results section, the anwer should be answered no. | Yes or no (1,0) |
| 1. Are the characteristics of the patients included in the study clearly described?  - In cohort studies and trials, inclusion and/or exclusion criteria should be given. In case-control studies, a case-definition and the source for controls should be given | Yes or no (1,0) |
| 1. Are the main findings of the study clearly described?  - Simple outcome data (including denominators and numerators) should be reported for all major findings so that the reader can check the major analyses and conclusions. (this question does not cover statistical tests which are considered below). | Yes or no (1,0) |
| 1. Does the study provide estimates of the random variability in the data for the main outcomes?  - In non normally distributed data the inter-quartile range of results should be reported. In normally distributed data the standard error, standard deviation or confidence intervals should be reported. If the distribution of the data is not described, it must be assumed that the estimates used were appropriate and the question should be answered yes | Yes or no (1,0) |
| 1. Have actual probability values been reported(e.g. 0.035 rather than <0.05) for the main outcomes except where the probability value is less than 0.001? | Yes or no (1,0) |
| 1. Were the subjects to participate in the study representative of the entire population from which they were recruited? | Yes or no (1,0); 0 if unable to determine |
| 1. Were those subjects who were prepared to participate representative of the entire population from which they were recruited?  - The proportion of those asked who agreed should be stated. Validation that the sample was representative would include demonstrating that the distribution of the main confounding factors was the same in the study sample and the source population | Yes or no (1,0); 0 if unable to determine |
| 1. Were the statistical tests used to assess the main outcomes appropriate?  - The statistical techniques used must be appropriate to the data. For example, nonparametric methods should be used for small sample sizes. Where little statistical analysis has been undertaken but where there is no evidence of bias, the question should be answered yes. If the distribution of the data (normal or not) is not described it must be assumed that the estimates used were appropriate and the question should be answered yes | Yes or no (1,0); 0 if unable to determine |
| 1. Were the main outcome measures used accurate (valid and reliable)  - For studies where the outcome measures are clearly described, the question should be answered yes. For studies which refer to other work or that demonstrates the outcome measures are accurate, the question should be answered as yes | Yes or no (1,0); 0 if unable to determine |

*descriptors from: Downs SH, Black N. The feasibility of creating a checklist for the assessment of the methodological quality both of randomised and non-randomised studies of health care interventions. J Epidemiol Community Health. 1998;52(6):377-84.

Appendix 4

| Author name and year | Downs and Black item 1 | Downs and Black item 2 | Downs and Black item 3 | Downs and Black item 4 | Downs and Black item 5 | Downs and Black item 6 | Downs and Black item 7 | Downs and Black item 8 | Downs and Black item 9 | Downs and Black item 10 | Total |
| --- | --- | --- | --- | --- | --- | --- | --- | --- | --- | --- | --- |
| Ahmad et al. (2015) | 1 | 1 | 1 | 1 | 1 | 1 | 1 | 1 | 1 | 1 | 10 |
| Balentine et al. (2010) | 1 | 1 | 1 | 1 | 0 | 1 | 1 | 1 | 1 | 1 | 9 |
| Batenburg (1997) | 1 | 1 | 1 | 0 | 1 | 1 | 1 | 1 | 1 | 1 | 9 |
| Batenburg et al. (1999) | 1 | 1 | 1 | 1 | 1 | 1 | 1 | 1 | 1 | 1 | 10 |
| Beach et al. (2007) | 1 | 1 | 1 | 1 | 1 | 1 | 1 | 1 | 1 | 1 | 10 |
| Bombeke et al. (2011) | 1 | 1 | 1 | 1 | 1 | 0 | 1 | 1 | 1 | 1 | 9 |
| Burnard & Morrison (1991) | 1 | 1 | 1 | 1 | 0 | 0 | 0 | 0 | 1 | 1 | 6 |
| Davis et al. (2006) | 1 | 1 | 1 | 1 | 1 | 1 | 0 | 1 | 1 | 1 | 9 |
| Davis et al. (2018) | 1 | 1 | 1 | 1 | 1 | 1 | 0 | 0 | 1 | 1 | 8 |
| Dockens et al. (2016) | 1 | 1 | 1 | 1 | 1 | 1 | 1 | 0 | 1 | 1 | 9 |
| El-Awaisi et al. (2017) | 1 | 1 | 1 | 1 | 1 | 1 | 0 | 1 | 1 | 1 | 9 |
| Fothan, Eshaq & Bakather (2019) | 1 | 1 | 1 | 1 | 1 | 1 | 1 | 1 | 1 | 1 | 10 |
| Gaufberg et al. (2018) | 1 | 1 | 1 | 1 | 1 | 1 | 1 | 1 | 1 | 1 | 10 |
| Grilo et al. (2013) | 1 | 1 | 1 | 1 | 1 | 1 | 1 | 1 | 1 | 1 | 10 |
| Haidet et al. (2001) | 1 | 1 | 1 | 1 | 1 | 0 | 0 | 0 | 1 | 1 | 7 |
| Haidet et al. (2002) | 1 | 1 | 1 | 1 | 1 | 0 | 1 | 1 | 1 | 1 | 9 |
| Hammerich et al. (2019) | 1 | 1 | 1 | 1 | 1 | 1 | 1 | 1 | 1 | 1 | 10 |
| Hardeman et al. (2015) | 1 | 1 | 1 | 1 | 0 | 1 | 1 | 1 | 1 | 1 | 9 |
| Harris et al. (2020) | 1 | 1 | 1 | 1 | 1 | 1 | 1 | 1 | 1 | 1 | 10 |
| Hauer et al. (2010) | 1 | 1 | 1 | 1 | 1 | 1 | 1 | 1 | 1 | 1 | 10 |
| Henschen et al. (2015) | 1 | 1 | 1 | 1 | 1 | 1 | 1 | 1 | 1 | 1 | 10 |
| Hirsh et al. (2012) | 1 | 1 | 1 | 1 | 0 | 1 | 0 | 0 | 1 | 1 | 7 |
| Hudson et al. (2016) | 1 | 1 | 1 | 1 | 1 | 1 | 1 | 1 | 1 | 1 | 10 |
| Hur, Cho & Choi (2017) | 1 | 1 | 1 | 1 | 1 | 1 | 1 | 0 | 1 | 1 | 9 |
| King & Violato (2020) | 1 | 1 | 1 | 1 | 1 | 1 | 1 | 1 | 1 | 1 | 10 |
| Krupat et al. (2009) | 1 | 1 | 1 | 1 | 0 | 1 | 0 | 0 | 1 | 1 | 7 |
| Lee et al. (2008) | 1 | 1 | 1 | 1 | 1 | 0 | 1 | 1 | 1 | 1 | 9 |
| Liu et al. (2019) | 1 | 1 | 1 | 1 | 1 | 1 | 1 | 1 | 1 | 1 | 10 |
| Madham, Rajpurohit & Gayathri (2010) | 1 | 1 | 1 | 1 | 1 | 0 | 1 | 1 | 1 | 1 | 9 |
| McNair et al. (2016) | 1 | 1 | 1 | 1 | 1 | 1 | 1 | 1 | 1 | 1 | 10 |
| Meirovich et al. (2016) | 1 | 1 | 1 | 1 | 1 | 0 | 0 | 1 | 1 | 1 | 8 |
| Michael, Dror & Miller (2019) | 1 | 1 | 1 | 1 | 1 | 0 | 1 | 0 | 1 | 1 | 8 |
| Mirsu-Pau, Tucker & Hardt (2012) | 1 | 1 | 1 | 1 | 1 | 0 | 1 | 1 | 1 | 1 | 9 |
| Moore (2009) | 1 | 1 | 1 | 1 | 1 | 0 | 0 | 0 | 1 | 1 | 7 |
| Mudiyanse et al (2015) | 1 | 1 | 1 | 1 | 1 | 0 | 1 | 0 | 1 | 1 | 8 |
| Noble et al. (2007) | 1 | 1 | 1 | 1 | 1 | 0 | 1 | 1 | 1 | 1 | 9 |
| Norris et al. (2015) | 1 | 1 | 1 | 1 | 1 | 1 | 1 | 1 | 1 | 1 | 10 |
| Pers et al. (2019) | 1 | 1 | 1 | 1 | 1 | 1 | 1 | 0 | 1 | 1 | 9 |
| Ribeiro, Krupat & Amaral (2007) | 1 | 1 | 1 | 1 | 1 | 1 | 1 | 1 | 1 | 1 | 10 |
| Rolfe (1994) | 1 | 1 | 1 | 1 | 1 | 1 | 1 | 1 | 0 | 1 | 9 |
| Rosewilliam et al. (2019) | 1 | 1 | 1 | 1 | 1 | 1 | 1 | 1 | 1 | 1 | 10 |
| Ross & Haidet (2011) | 1 | 1 | 1 | 1 | 1 | 0 | 1 | 1 | 1 | 1 | 9 |
| Ster et al. (2015) | 1 | 1 | 1 | 1 | 1 | 1 | 1 | 1 | 1 | 1 | 10 |
| Stoner et al. (2018) | 1 | 1 | 1 | 1 | 1 | 1 | 1 | 1 | 1 | 1 | 10 |
| Sweeney and Baker (2018) | 1 | 1 | 1 | 1 | 0 | 0 | 0 | 1 | 0 | 1 | 6 |
| Tsimtsiou et al. (2007) | 1 | 1 | 1 | 1 | 1 | 0 | 1 | 1 | 1 | 1 | 9 |
| Welch Bacon et al. (2018) | 1 | 1 | 1 | 1 | 1 | 0 | 1 | 1 | 1 | 1 | 9 |
| Zaudke et al. (2016) | 1 | 1 | 1 | 1 | 1 | 1 | 0 | 1 | 1 | 1 | 9 |
| Zeeni et al. (2016) | 1 | 1 | 1 | 1 | 1 | 1 | 1 | 1 | 1 | 1 | 10 |
